# Environmental and personal barriers encountered in community pharmacy settings based on public opinions

**DOI:** 10.2144/fsoa-2023-0166

**Published:** 2024-05-28

**Authors:** Ahmad Al-Azayzih, Walid Al-Qerem, Zelal Kharaba, Lara Albiss, Abdullah Alawneh, Dima Abu-Dalhoum, Laith Yousef, Mohammad Al-Amreen, Zeina Abedrabbo

**Affiliations:** 1 Department of Clinical Pharmacy, Faculty of Pharmacy, Jordan University of Science & Technology. P.O. Box 3030, Irbid, 22110, Jordan; 2 Faculty of Pharmacy, Al-Zaytoonah University of Jordan, Amman, 11733, Jordan; 3 College of Pharmacy, AL Ain University, Abu Dhabi, United Arab Emirates; 4 Honorary Associate Lecturer, Faculty of Medical Sciences, Newcastle University, Newcastle Upon Tyne, UK

**Keywords:** community pharmacy, environmental barrier, personal barrier, pharmacist

## Abstract

**Aim:** We aimed in this study to assess the potential environmental and personal barriers encountered in the community pharmacies in Jordan. **Methods:** A validated self-administered survey was distributed online to 721 participants from all regions in Jordan. **Results:** All approached participants (721 subjects) answered the survey. The most common environmental and personal barriers reported were that community pharmacies were not disabled patients friendly (59.4%) and pharmacist's low self-confidence (80.4%), respectively. Socioeconomic characteristics such as being male, married, receiving lower income and having higher educational degrees, were associated with an increase in reported personal communication barriers. **Conclusion:** Our study indicated that environmental and personal barriers are prevalent in the community pharmacy practice, which could impact the quality of pharmaceutical services provided.

## Plain language summary

This study aimed to evaluate the environmental and personal barriers encountered in the community pharmacy settings in Jordan through utilizing a validated online self-administered survey that was distributed to 721 participants from all areas in Jordan. The study found that participants median age of 30 (28–33) years old and the most common physical and personal barriers reported were that community pharmacies were not friendly for patients with disability (59.4%) and pharmacist's low self-confidence (80.4%), respectively. This study concluded that both physical and personal barriers are prevalent in the community pharmacy practice.

## Abstract (plain language summary)

This study aimed to assess the potential environmental and personal barriers encountered in the community pharmacies in Jordan.

Responses from participants was collected utilizing a validated self-administered survey which distributed online.

A total of 721 subjects participated in this study from all regions in Jordan.

The most common environmental barriers reported were that community pharmacies were not disabled patients friendly (59.4%) followed by the prescription counter was inappropriate for communication (43.3%).

The most reported personal barrier was appeared pharmacist's low self-confidence (80.4%).

The most reported reason to consult or visit pharmacy was dispensing medications (54.65%) followed by purchasing over the counter (OTC) medications (51.46%).

The most reported reasons for favoring a specific community pharmacy over the others was the proximity of pharmacy location (75.87%) followed by pharmacist knowledge and personality (63.66%).

Males exhibit a higher likelihood of belonging to the high personal barrier group in comparison to females.

Married participants demonstrate increased odds of being in the high personal barrier group when compared with unmarried participants.

Participants with lower monthly income are more likely to fall in the higher personal barrier group.

Participants with a diploma degree are less likely to be part of the high personal barrier group when compared with those with a graduate degree (master's or Ph.D.).

This study indicated that environmental and personal barriers are prevalent in the community pharmacy practice.

In-depth evaluation of personal and environmental barriers and their predictors is warranted to improve communication quality and therefore the pharmaceutical services provided by community pharmacy settings in Jordan.

Community pharmacies are considered to be fundamental contributors to healthcare systems worldwide. Most of the public seek the services of community pharmacies, including medication dispensing, consultation services for minor health problems such as management of cold or flu, over the counter supplements and soliciting advice regarding various health-related issues [^1-4^].

Services provided by community pharmacies have changed over the past few years. While, decades ago, community pharmacist duties were limited to the dispensation of patient prescriptions and extemporaneous compounding of drug mixtures by pharmacists, pharmacy services have advanced rapidly and modern community pharmacists are engaged in a wider range of tasks. These tasks include more roles in patient therapeutic plan improvements, assessing drug–drug interactions and helping to avoid potential harm to customer patients. Community pharmacists may also provide vaccinations [5], point of care testing [6], smoking cessation programs [7], helping patients to reach healthier lifestyles and better quality of life [8,9], and optimizing patient medication therapy [10].

As counseling patients about their health and medications is one of the essential roles for community pharmacists, high-quality communication between pharmacists and their patients is a keystone in the process of effective medical advice in order to reach the best health outcomes expected from patients [1]. Pharmacist–patient communication quality could be affected by a range of barriers involving physical barriers related to community pharmacy design and layout, personal barriers linked to pharmacist personality [11,12], cultural barriers [13,14] and language barriers [15].

In Jordan, similar to other world regions, community pharmacies are considered to be the first health services approached by most patients, due to ease of access to pharmacists and their availability without needing scheduled appointments, their widespread occurrence and proximity across the county, and availability during weekends and holidays.

As there is a continuous demand to keep up with challenges that face health services provided by community pharmacies, it is crucial to identify the barriers that might hamper effective communication between pharmacists and patients, hindering attempts to provide better care and higher satisfaction. Furthermore, effective communication necessitates resolving all potential personal and physical barriers encountered by patients during their visit to community pharmacies, this will improve the rational use of medication by patients and ensure patient adherence to medication therapy through effective counseling and education. Thus, this study aims to examine all potential physical and personal barriers that might affect pharmacy services from the perspective of patients, and their suggestions for improvements.

## Methods

### Ethical approval

Ethical Approval to conduct the study was obtained from the Institutional Review Board and deanship of research at Jordan University of Science and Technology and its teaching hospital, the King Abdulla University Hospital (KAUH). Ethical Approval number is 30/161/2023.

### Study design & subjects

This study was a cross sectional self-administered survey completed online through google forms. A link including the survey questions was sent to all potential participants from the public across Jordan. The participant's included age was ≥18 years old. The first page of the survey stated that participation in the study is voluntary, and all data will be kept confidential and will be utilized only for scientific research goals. The survey did not ask for names or any identifying information from the participants.

### Study instrument

A detailed search through the literature [1,11,12,^16-19^] was conducted to identify major physical and personal barriers encountered by the public during visits to community pharmacies. Authors structured the survey questions carefully in clear understandable language, taking into consideration the differences in backgrounds and education of all participants. The survey was distributed to ten subjects to ensure question readability and patient comprehension, validity of the survey, and average completion time. The pilot sample raised comments and feedback was then taken into consideration to improve the survey quality. The pilot sample responses were not included in the final statistical analysis. The survey was divided into four domains: the first domain evaluated the age, gender, educational level, sociodemographic and socio-economic status of the participants. The second domain focus was to identify the potential physical/environmental barriers might affect quality of pharmaceutical services provided by community pharmacies. The second domain included 11 questions covering topics such as the availability of private counseling area, decoration and design of the pharmacy, crowdedness of the dispensing area and elevation of the dispensing counter, waiting area comfort and availability, visual and hearing acuity in the community pharmacy, accessibility for patients with special need such as geriatrics and disabled patients. The third domain identified the potential personal barriers (pharmacist personality) which could hinder the provision of optimum services. The third domain consisted of five questions regarding pharmacist confidence levels during consultation, personality and approachability of pharmacists, pharmacist's knowledge, willingness to help with medication inquires, shyness of the pharmacist, easiness and ability to discuss sensitive health-related issues with pharmacists, need to talk to pharmacist through third parties (such as pharmacist technician or pharmacy employees). The fourth domain evaluated the reasons behind patients' preference to prefer specific pharmacy to visit and main reasons leading them to consult community pharmacies.

### Sampling technique & sample size calculation

A convenience sampling method was utilized involving the selection of study participants based on convenience to the investigators and availability and willingness of subjects to participate. With margin of error of 5%, confidence level of 95%, and unlimited population size, the ideal required sample size was equal to 385 subjects [20].

### Statistical analysis

SPSS version 28 was employed for data analysis. Categorical variables were presented as frequency (%) and continuous variables as median (95%Cl.). Cronbach's alpha was calculated to evaluate the internal consistency of the developed latent variables (Cronbach's alpha for the environmental barriers = 0.71, Cronbach's alpha for the personal barriers = 0.70). Personal level score was computed by summing the points for each designated item, Subsequently, participants were divided into high and low groups based on their scores. Those individuals whose scores exceeded the median value were classified into the high group, while those with scores lower than the median were categorized into the low group. A binary regression model was built to investigate the relationship between sociodemographic characteristics and personal barriers. The independent variables include age, gender, marital status, monthly income, level of education, type of pharmacy to get medical advice, age of pharmacists that participants generally interact with, and pharmacy location. A significant level was determined at p < 0.05.

## Results

### Sociodemographic characteristics of the enrolled participants

All approached participants (721 subjects) answered the survey. The present study revealed that 65% of the participants were females with a median age of 30 (28–33) years old. More than half of the participants had a bachelor's degree (67.4%) and were earning less than 500 JOD/month (55.9%). Many of the enrolled participants were seeking independent pharmacy to get their medication, or medical advice (65.37%) (Table 1).

**Table 1.** Sociodemographic characteristics of the enrolled participants.

		Frequency (%) or Median (95Cl)
Age		30 (28–33)
Gender	Male	252 (35%)
	Female	469 (65%)
Marital status	Married	354 (49.1%)
	Non married	367 (50.9%)
Monthly Income	Less than 500 JOD	403 (55.9%)
	500–1000JOD	229 (31.8%)
	More than 1000 JOD	89 (12.3%)
Level of education	High school or less	82 (11.4%)
	Diploma	71 (9.8%)
	Bachelors	486 (67.4%)
	Master, PhD	82 (11.4%)
What is the type of pharmacy you seek to get your medication or seek medical advice, most of the time?	Chain Pharmacy	247 (34.3%)
	Independent pharmacy	474 (65.7%)
Pharmacy location	North	190 (26.6%)
	Middle	253 (35.4%)
	South	272 (38%)
In general what age range does the pharmacist that you interact with belong to?	Recent graduates (20s)	116 (16.1%)
	Mid-career professionals (30s and 40s)	451 (62.6%)
	Experienced (50s or older)	154 (21.4%)

### Environmental barriers

Table 2 demonstrates participants responses to the environmental barriers against good pharmaceutical services, the results revealed that the most reported barriers were ‘The pharmacy was not disability friendly’, ‘The prescription counter separating patients from the pharmacy personnel is inappropriate for effective communication’ and ‘There was a lack of privacy area (counseling area)’ (59.4%, 43.3%, and 39% respectively). On the other hand, the least reported barrier was ‘The pharmacist was not always visible during your last visit’ (3.1%) followed by ‘Inappropriate pharmacy temperature’ (3.3%) and ‘Inappropriate light, visibility, or poor visual quality’ (6%).

**Table 2.** Environmental barriers.

Barrier	No frequency (%)	Yes frequency (%)
The prescription counter separating patients from the pharmacy personnel is inappropriate for effective communication?	409 (56.7)	312 (43.3)
Is it crowded, noisy there at prescription area?	488 (67.7)	233 (32.3)
There was a lack of privacy area (counseling area)	440 (61)	281 (39)
Inappropriate light, visibility, or poor visual quality	678 (94)	43 (6)
Inappropriate pharmacy temperature	697 (96.7)	24 (3.3)
Uncomfortable Pharmacy design and decoration	622 (86.3)	99 (13.7)
The waiting area was uncomfortable	480 (66.6)	241 (33.4)
It was not easy for me to access the pharmacist to have a meaningful dialogue	598 (82.9)	123 (17.1)
The pharmacist was not always visible during your last visit	699 (96.9)	22 (3.1)
Was it hard to get the pharmacist's attention in your last visit?	661 (91.7)	60 (8.3)
The pharmacy was not disability friendly	293 (40.6)	428 (59.4)

### Personal barriers

Participants' responses to the personal barriers are presented in (Table 3). The most reported barrier was appeared pharmacist's low self-confidence, which was agreed/strongly agreed by 80.4%, while the least reported barriers were pharmacist's lack of interest in answering patients' questions (9%) and appeared pharmacist's shyness (9.1%).

**Table 3.** Personal barriers.

Barrier	Strongly disagree Frequency (%)	Disagree Frequency (%)	Neutral Frequency (%)	Agree Frequency (%)	Strongly agree Frequency (%)	Median (95% Cl)
The pharmacist seems to have low self-confidence	6 (0.8%)	9 (1.2%)	126 (17.5%)	382 (53%)	198 (27.5%)	4 (4–5)
In my opinion, the pharmacist I dealt with in my last visit seems shy	105 (14.6%)	347 (48.1%)	203 (28.2%)	55 (7.6%)	11 (1.5%)	2 (2–3)
I felt discomfort in discussing sensitive situations/issues with the pharmacist	92 (12.8%)	279 (38.7%)	201 (27.9%)	113 (15.7%)	36 (5%)	2 (2–3)
In my opinion, the pharmacist was not knowledgeable enough to answer my questions	124 (17.2%)	385 (53.4%)	137 (19%)	57 (7.9%)	18 (2.5%)	2 (2–3)
In my opinion, the pharmacist showed a lack of interest in answering my questions in my last visit	131 (18.2%)	413 (57.3%)	112 (15.5%)	45 (6.2%)	20 (2.8%)	2 (2–3)

### Reasons to consult or visit community pharmacy

The most reported reason to consult or visit pharmacy was dispensing medications (54.65%) followed by purchasing over the counter (OTC) medications (51.46%). On the other hand, health screenings were the least reported reason (11.51%) (Table 4).

**Table 4.** Reasons to consult or visit community pharmacy.

Reason	Frequency (%)
Refill regular medications	179 (24.83%)
Dispensing medications	394 (54.65%)
Purchase over the counter (OTC) medications	371 (51.46%)
Seek advice and consultations from pharmacists	287 (39.81%)
Health screenings such as blood pressure checks, blood glucose monitoring, pulse oximeter	83 (11.51%)

### Reasons to prefer one pharmacy over the other

Reasons to prefer one pharmacy over the other are presented in (Table 5), and the most reported reason was the proximity of pharmacy location followed by pharmacist knowledge and personality (75.87% and 63.66%, respectively), while the least reported reasons were additional services provided (13.59%) and short waiting time (14.42%).

**Table 5.** Reasons to prefer one pharmacy over the other.

Reason	Frequency (%)
Nearby location	547 (75.87%)
Personality and knowledge of pharmacist	459 (63.66%)
Short waiting times	104 (14.42%)
Additional services (blood pressure, glucose, pulse measures)	98 (13.59%)
Insurance coverage	170 (23.58%)

### Binary regression between sociodemographic characteristics & personal barriers

A binary regression analysis was conducted to investigate the relationship between sociodemographic characteristics and personal barriers. The findings revealed that males exhibit a higher likelihood of belonging to the high personal barrier group in comparison to females (OR: 1.419, 95% CI: 1.013–1.987, p = 0.042). Additionally, married participants demonstrate increased odds of being in the high personal barrier group when compared with unmarried participants (OR: 1.609, 95% CI: 1.015–2.550, p = 0.043). On the other hand, participants with a monthly income exceeding 1000 JOD are less likely to fall into the high personal barrier category than those earning less than 500 JOD monthly (OR: 0.505, 95% CI: 0.288–0.886, p = 0.017). Moreover, individuals with a diploma degree are less likely to be part of the high personal barrier group when compared with those with a graduate degree (master's or Ph.D.) (OR: 0.489, 95% CI: 0.244–0.980, p = 0.044) (Table 6).

**Table 6.** A binary regression between sociodemographic characteristics and personal barriers.

Characteristics		p-value	OR	95% C.I. for OR	
				Lower	Upper
Age		0.699	0.996	0.978	1.015
Gender	Male	0.042	1.419	1.013	1.987
	Female	(REF)	(REF)	(REF)	(REF)
Marital status	Married	0.043	1.609	1.015	2.550
	Non married	(REF)	(REF)	(REF)	(REF)
Monthly Income	500 JOD-1000JOD	0.814	1.047	0.716	1.531
	More than 1000 JOD	0.017	0.505	0.288	0.886
	Less than 500 JOD	(REF)	(REF)	(REF)	(REF)
Level of education	High school or less	0.419	0.763	0.396	1.470
	Diploma	0.044	0.489	0.244	0.980
	Bachelors	0.737	0.917	0.554	1.518
	Master, PhD	(REF)	(REF)	(REF)	(REF)
Type of pharmacy to get medical advice	Chain Pharmacy	0.076	1.364	0.969	1.920
	Independent pharmacy	(REF)	(REF)	(REF)	(REF)
Age of pharmacists that participants generally interact with	Recent graduates (20s)	0.510	1.184	0.717	1.955
	Mid-career professionals (30s and 40s)	0.249	1.255	0.853	1.848
	experienced (50s or older)	(REF)	(REF)	(REF)	(REF)
Pharmacy location	North	0.227	0.762	0.491	1.183
	Middle	0.525	0.878	0.589	1.311
	South	(REF)	(REF)	(REF)	(REF)

## Discussion

Our present study, to best of our knowledge is the first to focus mainly on identifying both personal and environmental barriers against good pharmacy services and effective communication between customers and pharmacists in the community pharmacy settings.

In the present study, participants identified ‘The pharmacy was not disability friendly’ as the main environmental barriers they encounter especially when they accompany their relatives or friends suffering from physical disability. A recent editorial letter indicated that physical layout of the community pharmacies as one of the main obstacles facing disabled peoples and hindering their access to community pharmacies services [12]. Another study indicated that public perception toward community pharmacies accessibility were problematic for many reasons including lack of wheelchair path to community pharmacies entrance, slippery ceramic floors and risk of falls, and sparse waiting spots in the community pharmacies area [11].

Other common environmental barriers revealed in participants responses were appropriateness of the prescription counter separating patients from the pharmacy personnel for effective communication, and lack of privacy area (counseling area). These results were in concordance with previous reports from Jordan and other regions. A recent study showed that lack of privacy in community pharmacy settings is among the most common barriers encountered during consultation with pharmacist [16]. A qualitative study indicated that community pharmacies environments were inconvenient for people to obtain pharmacist advice on private and sensitive issues [11]. Prescription counter presence is crucial in community pharmacies settings to separate prescription drugs behind the counter, give the hospital and community pharmacist the necessary space to work efficiently, and in addition to other social distancing measurements to minimize the risk of contracting airborne infections in both pharmacists and patients especially during pandemics such as COVID-19 pandemic and seasonal influenzas outbreaks [21]. On the other hand, it is also critical to meet proper conditions to provide enough space for pharmacist to work freely and remain clear at all times during the processing and dispensing of prescriptions, without affecting the efficacy and quality of communications between pharmacist and visiting customers.

The most common personal barrier reported by this study participants was pharmacist's low self-confidence. An exploratory study aimed to identify reasons behind lacking either responsibility and/or confidence in different pharmacy practice environments concluded that six potential barriers could inhibit the development of self-confidence and sense of responsibility among pharmacists [19]. These barriers include pharmacists' beliefs that they have no place in medical hierarchy, and so, cannot take responsibility for patients since they cannot prescribe medications. Other barriers which could lead to pharmacist's low self-confidence, the pharmacist believes that they are not ready to take responsibility for making decisions concerning clinical aspects of their profession, and concern about public perceptions identifying pharmacist main role as medications dispensers.

The most common reasons to seek community pharmacy services were dispensing medications followed by obtaining over the counter (OTC) supplements. These results were in harmony with previous studies findings from Jordan and Qatar concluding that prescription dispensing and obtaining OTC medications were the most common reasons behind visiting community pharmacies [16,17,22].

Health screenings such as blood pressure checks, blood glucose monitoring, pulse oximeter was the least reported response among reasons to visit community pharmacies. This option low response rate might be justified due to number of factors including poor interest among pharmacists in integrating health screening services into their provided services, also, it might be related to patients' perception and believes that even simple screening health process should be recommended by physicians and under their supervision. A recent study indicated that only 54.1% of the community pharmacists believed that promoting health services would be reflected positively on their patient's health [23].

Proximity of pharmacy location followed by pharmacist knowledge and personality were the most common responses reported in this study regarding preference of one community pharmacy over the other. A nearby pharmacy location was reported in other studies as the main reason for choosing any community pharmacy over the other followed by available range of products and services in the community pharmacies [16,17].

Regression model has revealed that socioeconomic characteristics such as being male, married, receiving lower income, and having higher educational degrees could increase the community pharmacies customers likelihood of reporting more personal communication barriers compared with their counterparts. Such correlation could be explained by insufficient health literacy existed among those with lower monthly income and higher level of expectations from those holding advanced educational degrees about the quality of pharmaceutical information and counseling services provided by community pharmacists which might not be satisfactory for many of them. A previous study showed that patients with poor health literacy reported less satisfaction level for the communication quality between pharmacist and patients on the personal communication parameters such as clarity of medical information provided and perceived appropriate response to patients interests and concerns [24].

This study identified number of environmental and personal barriers encountered by public during their consultation of pharmaceutical services provided by community pharmacies in Jordan. Such barriers could be solved by implementing new regulatory approaches to promote community pharmacies roles in society and encourage the pharmacists to expand their services beyond drug dispensing and OTC products promotion.

This study is limited by the nature of the self-administered questionnaire, which carries social desirability bias where participants could answer the question based on the favorable belief of the society, but not themselves.

## Conclusion

Our study revealed that various environmental and personal barriers could impair the effective communication between pharmacists and visitors to community pharmacies. The most common believed environmental barriers were that community pharmacies were not disability friendly, followed by inappropriate prescription counter design and elevation impacting effective communication between pharmacist and patients. The most believed personal barrier reported was appeared pharmacist's low self-confidence. These results undoubtedly raised the need for changing the community pharmacy practice through resolving these barriers and reaching better quality of communication between persons who seek pharmacy services and pharmacists.

## Recommendations

Future studies should be conducted to evaluate the personal and environmental barriers as well as other barriers associated with poor communication quality, and the quality of pharmaceutical service provided in general. Better understanding of these barriers would help in solving these barriers and hence, improving the quality of health services provided by community pharmacy settings.
